# Tetraplegia is associated with increased hypoxic ventilatory response during nonrapid eye movement sleep

**DOI:** 10.14814/phy2.15455

**Published:** 2022-09-06

**Authors:** Sarah Vaughan, Abdulghani Sankari, Sean Carroll, Mehdi Eshraghi, Harold Obiakor, Hossein Yarandi, Susmita Chowdhuri, Anan Salloum, M. Safwan Badr

**Affiliations:** ^1^ Department of Medicine John D. Dingell VA Medical Center Detroit Michigan USA; ^2^ Department of Internal Medicine Wayne State University Detroit Michigan USA; ^3^ Department of Medical Education Ascension Providence Hospital Southfield Michigan USA

**Keywords:** acute intermittent hypoxia, hypoxia, long‐term facilitation, plasticity, spinal cord injury

## Abstract

People with cervical spinal cord injury (SCI) are likely to experience chronic intermittent hypoxia while sleeping. The physiological effects of intermittent hypoxia on the respiratory system during spontaneous sleep in individuals with chronic cervical SCI are unknown. We hypothesized that individuals with cervical SCI would demonstrate higher short‐ and long‐term ventilatory responses to acute intermittent hypoxia (AIH) exposure than individuals with thoracic SCI during sleep. Twenty participants (10 with cervical SCI [9 male] and 10 with thoracic SCI [6 male]) underwent an AIH and sham protocol during sleep. During the AIH protocol, each participant experienced 15 episodes of isocapnic hypoxia using mixed gases of 100% nitrogen (N_2_) and 40% carbon dioxide (CO_2_) to achieve an oxygen saturation of less than 90%. This was followed by two breaths of 100% oxygen (O_2_). Measurements were collected before, during, and 40 min after the AIH protocol to obtain ventilatory data. During the sham protocol, participants breathed room air for the same amount of time that elapsed during the AIH protocol and at approximately the same time of night. Hypoxic ventilatory response (HVR) during the AIH protocol was significantly higher in participants with cervical SCI than those with thoracic SCI. There was no significant difference in minute ventilation (*V*_.E._), tidal volume (*V*_.T._), or respiratory frequency (*f*) during the recovery period after AIH in cervical SCI compared to thoracic SCI groups. Individuals with cervical SCI demonstrated a significant short‐term increase in HVR compared to thoracic SCI. However, there was no evidence of ventilatory long‐term facilitation following AIH in either group.

## Graphical abstract

This study compares the ventilatory responses to acute intermittent hypoxia during sleep in two groups of volunteers with two levels of spinal cord injury (SCI) cervical versus high thoracic. The study reports for the first time the enhanced short‐term hypoxic ventilatory response in cervical SCI during natural sleep but without significant long‐term ventilatory plasticity. Our findings have several potential clinical implications related to the pathogenesis and treatment of sleep‐disordered breathing in individuals with SCI, especially cervical SCI.

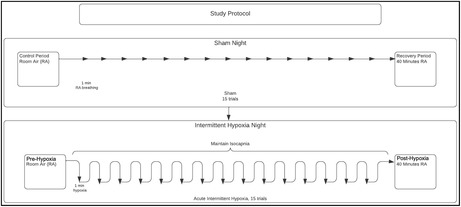

## 1 INTRODUCTION

It is estimated that there are approximately 282,000 people in the United States living with spinal cord injury (SCI), with 17,000 new cases each year (National Spinal Cord Injury Statistical Center, 2016). Respiratory dysfunction is a major cause of morbidity and mortality in people with SCI, particularly in cervical injury levels (Daoud et al., 2020). Individuals with cervical SCI experience chronic intermittent hypoxia (CIH) secondary to multiple factors, including abnormal chest wall mechanics, decreased lung volume, impaired respiratory muscles, and disordered breathing during sleep (Bascom et al., 2015; Brown et al., 2006; Daoud et al., 2020; Sankari, Bascom, Chowdhuri, et al., 2014; Sankari, Bascom, Oomman, et al., 2014; Sankari, Martin, et al., 2015; Scanlon et al., 1989). Conversely, respiratory plasticity in response to CIH may contribute to respiratory recovery following cervical SCI (Ling et al., 2001).

Two types of respiratory plasticity, the hypoxic ventilatory response (HVR) and ventilatory long‐term facilitation (LTF), are induced by exposure to acute intermittent hypoxia (AIH; Lee et al., 2009; Mateika & Narwani, 2009; Powell et al., 1998). HVR takes place during exposure to hypoxia and is represented by a gradual increase in minute ventilation from the initial hypoxic episode to the final hypoxic episode (Gerst et al., 2011; Lee et al., 2009; Powell et al., 1998; Wadhwa et al., 2008). LTF is a form of respiratory plasticity where exposure to AIH leads to a sustained increase in ventilatory motor output after the termination of hypoxic exposure. LTF has been demonstrated in several animal models and humans during sleep (Babcock & Badr, 1998; Babcock et al., 2003; Cao et al., 1992; Chowdhuri et al., 2008; Feldman et al., 2003; Fregosi & Mitchell, 1994; Millhorn et al., 1980; Mitchell et al., 2001; Olson et al., 2001; Pierchala et al., 2008; Shkoukani et al., 2002; Vinit et al., 2009). Interestingly, individuals living with cervical SCI demonstrated enhanced ventilatory LTF following episodic isocapnic hypoxia during wakefulness (Sankari, Bascom, et al., 2015; Tester et al., 2014). Likewise, the presence of sleep‐disordered breathing (SDB) has been positively linked to hypoxia‐induced motor recovery in individuals with SCI (Vivodtzev et al., 2020). One potential explanation is that prior exposure to CIH may promote ventilatory LTF following AIH, likely through a combination of activation of peripheral and central pathways (Flavell et al., 1992; Klefbeck et al., 1998; Peng et al., 2003; Salloum et al., 2010). A recent study in healthy adults showed that episodes of hyperoxia‐induced ventilatory inhibition play a role in attenuating the ventilatory LTF during the recovery period. This suggests an important role for not only peripheral chemoreflex but also the central chemoresponses to AIH (Vermeulen et al., 2020).

Understanding the relative contribution of chemosensitivity to hypoxia in individuals with SCI is important for identifying the responsible mechanism for unstable breathing during sleep. We have previously demonstrated that the injury level may influence ventilatory responses following AIH during wakefulness, being more robust in individuals with cervical relative to thoracic SCI (Sankari, Bascom, et al., 2015). However, no previous studies assessed the effect of AIH during sleep in individuals with SCI. Therefore, we hypothesized that individuals with cervical SCI would demonstrate potentiated HVR and LTF in response to AIH exposure compared to individuals with thoracic SCI during sleep.

## 2 METHODS

### 2.1 Participants

The Human Investigation Committees of the Wayne State University School of Medicine and the John D. Dingell Veterans Affairs Medical Center approved the experimental protocols. This study was registered on Clinicaltrials.gov as NCT02922894. Written informed consent was obtained from all participating subjects. We studied 20 adult participants with chronic traumatic SCI (at least 8 months after injury), including 10 with cervical injuries (C4–C6) and 10 with thoracic injuries (T2–T6) with American Spinal Injury Association (ASIA) scores A, B, C, or D. Participant level demographics are presented in Table 1. The study was designed to include 10 participants with cervical level injuries and 10 participants with high thoracic level injuries.

**TABLE 1:** Participant characteristics by group

	All	Cervical	Thoracic
Number	20	10	10
Age (years)	48.9 ± 14.5	51.8 ± 15.6	46.0 ± 13.3
BMI (kg/m^2^)	27.8 ± 4.8	26.2 ± 5.4	29.4 ± 3.8
Gender (M/F)	15/5	9/1	6/4
AHI (events/h) range	25.1 ± 20.0 1.2–72.2	25.8 ± 17.4 6.8–59.8	24.4 ± 23.1 1.2–72.2
Mild SDB (<15 events/h) *N* (%)	9 (45)	4 (40)	5 (50)
Moderate/severe SDB (≥15 events/h) *N* (%)	11 (55)	6 (60)	5 (50)
ODI (events/h) IQ Range	10.4 ± 15.2 0–58.7	9.5 ± 11.9 0.9–38.5	11.3 ± 18.6 0–58.7

*Note*: Cervical and thoracic refer to the level of spinal cord injury. Data are presented as the mean ± SD. There were no significant differences between the cervical and thoracic groups with regard to age (*p* = 0.38), BMI (*p* = 0.14), AHI (*p* = 0.88), or ODI (*p* = 0.80) by independent *t* test.

Abbreviations: AHI, apnea‐hypopnea index; BMI, body mass index; IQ, interquartile range; ODI, oxygen desaturation index; SDB, sleep‐disordered breathing.

### 2.2 Breathing circuit

Each participant was connected to a breathing circuit with an appropriate‐sized, airtight silicone nasal mask (Philips Respironics). Full‐face masks (ResMed) were used if nasal masks were unsuccessful due to the inability to prevent mouth breathing during data collection. The presence of mouth breathing was detected using a high level of monitoring for a leak in the signals. The leak was addressed during data collection by the use of chin straps and mouth tape. The mask was connected to a Plateau Exhalation Valve (Philips Respironics) via a pnemotachometer (model 3700A; Hans Rudolph). The valve served as an exhaust vent by providing a continuous leak path in the breathing circuit. Three cylinders containing 100% oxygen (O_2_), 100% nitrogen (N_2_), and 40% carbon dioxide (CO_2_) balanced with N_2_ were connected to the inspiratory circuit through a gas blender (model PMR4) and used according to the protocol stated below. End‐tidal CO_2_ (P_ET_CO_2_) and O_2_ (P_ET_O_2_) were measured with gas analyzers (GEMINI, CWE Inc.). These methods have been previously described (Chowdhuri et al., 2015; Sankari, Bascom, et al., 2015).

### 2.3 Measurements

Attended full‐polysomnography (PSG) studies were obtained from all participants using a SomnoStar PSG sleep system (Vyaire Medical). The SomnoStar PSG sleep system meets the American Academy of Sleep Medicine (AASM) criteria for PSG and has a sampling rate of 500 Hz. AHI is determined by calculating the average number of apneas/hypopneas per hour. ODI is calculated as the average number of desaturations per hour, where the mean oxygen saturation (P_ET_O_2_) decreases by at least 3% during sleep. Inspiratory airflow was measured with a pneumotachometer (model 3700A; Hans Rudolph) attached to an RSS 100 HR Pneumotach System (Hans Rudolph). The tidal volume (*V*_.T._) was obtained from the electronic integration of the flow signal. Signals were recorded using a PowerLab data acquisition system (ADInstruments) and displayed using LabChart software (ADInstruments). Arterial O_2_ saturation (SaO_2_) was measured by a pulse oximeter (Biox 3740; Ohmeda Medical).

### 2.4 Acute intermittent hypoxia

**FIGURE 1:**
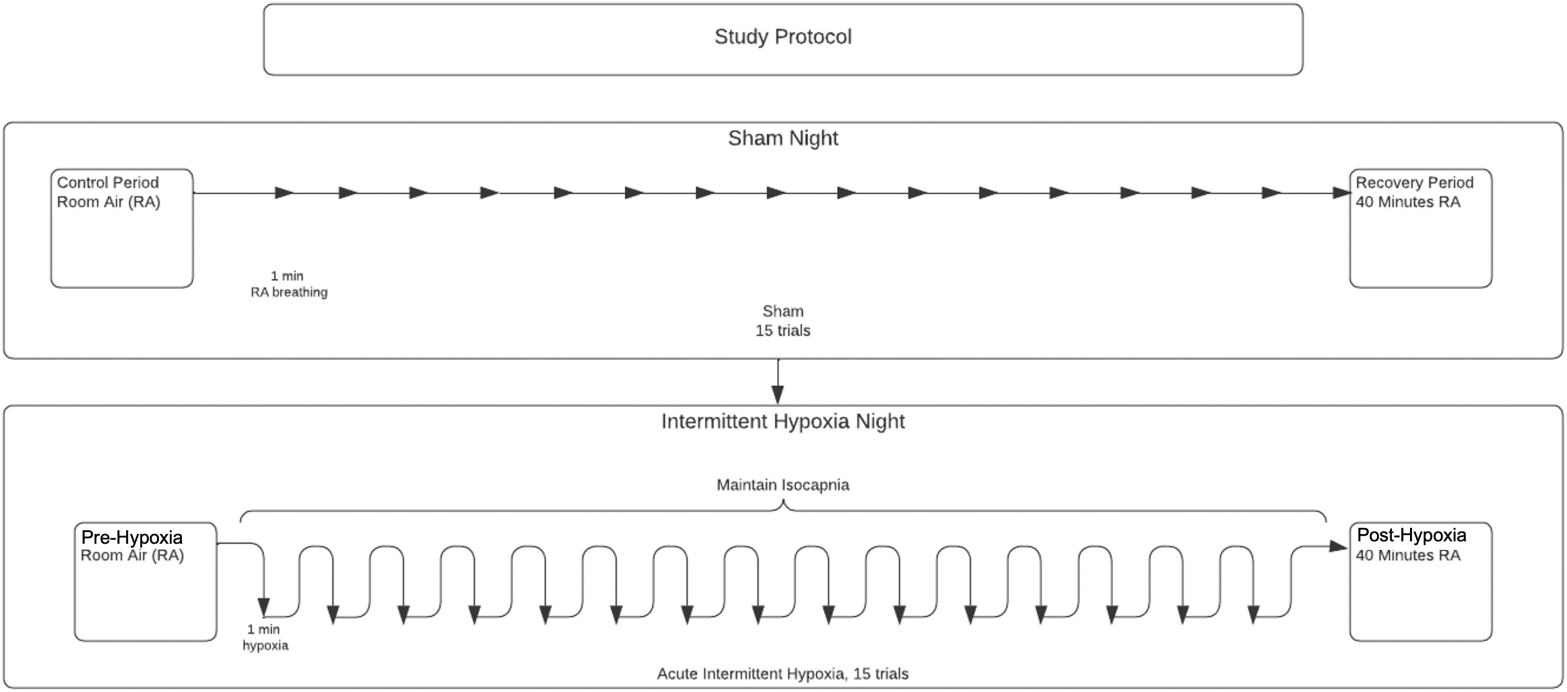
A diagram of the study protocol.

**FIGURE 2:**
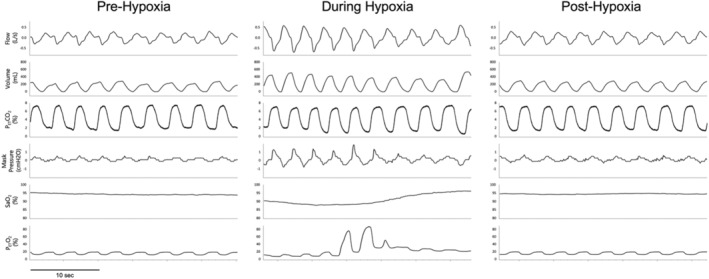
A representative polygraph recording of the intermittent hypoxia protocol illustrates respiratory changes and demonstrates that isocapnia was maintained during the AIH episodes. This tracing is from a female participant with a thoracic spinal cord injury. Her apnea‐hypopnea index was 8.6, and her oxygen desaturation index was 3.0.

All participants experienced AIH and sham protocols on two separate occasions during their standard nocturnal sleep period, using the same procedure and time control. All trials were conducted during stable nonrapid eye movement (NREM) sleep. We chose NREM sleep as adults spend most of their spontaneous sleep in this stage, which allows for the completion of repeated assessments at different points from the same night. For the baseline control period, participants breathed room air for 15 min during NREM sleep. This was followed by 15 episodes of hypoxia lasting 1 min each. Hypoxia was terminated abruptly with two breaths of 100% O_2_. Once O_2_ saturation returned to baseline levels, there was a short recovery period of approximately 1 min on room air before the next episode of AIH (Figure 1). Hypoxia was induced by adding mixed gases of 100% nitrogen (N_2_) and 40% carbon dioxide (CO_2_) to the breathing circuit to achieve P_ET_O_2_ of less than 90%. During the induction of hypoxia, the average inspired O_2_ was 10.71%. Isocapnia was maintained at a P_ET_CO_2_ of approximately 43 mmHg for individuals with cervical SCI and 41 mmHg for individuals with thoracic SCI (Table 2) throughout the AIH episodes by bleeding CO_2_ into the circuit from the blender and manually adjusting the flow. Participants are expected to be asleep throughout the entire hypoxia and recovery protocols; however, occasional arousals can occur in response to AIH. Therefore, hypoxic episodes in which arousals occurred were considered unsuccessful and additional episodes were performed until 15 successful episodes were completed. If the participant woke up, had arousal, entered into REM sleep, or experienced nonstable sleep for longer than 20 min, the entire protocol was repeated from the beginning. The number of AIH episodes experienced by each participant during the intervention night is listed in Table 4. A representative polygraph recording showing an expanded trace of the AIH protocol demonstrates that isocapnia was maintained throughout the AIH episodes and is presented in Figure 2.

**TABLE 2:** Effect of acute intermittent hypoxia on ventilation

	Cervical			Thoracic		
	Pre‐hypoxia	During hypoxia	Post‐hypoxia	Pre‐hypoxia	During hypoxia	Post‐hypoxia
*V*_.E._ (L/min)	7.5 ± 2.6	11.5 ± 3.3*	6.9 ± 1.7	6.5 ± 2.6	10.5 ± 4.6*	6.4 ± 2.7
*V*_.T._ (L)	0.5 ± 0.2	0.7 ± 0.2*	0.5 ± 0.1	0.4 ± 0.1	0.6 ± 0.2*	0.4 ± 0.1
*f* (BPM)	14.0 ± 3.0	16.5 ± 4.2*	13.7 ± 3.0	15.9 ± 4.3	18.5 ± 4.5*	16.1 ± 5.4
SaO_2_ (%)	96.1 ± 2.3	88.9 ± 2.5*	95.9 ± 1.8	95.6 ± 2.9	87.5 ± 2.7*	95.9 ± 1.4
P_ET_CO_2_	43.2 ± 5.0	43.9 ± 5.1	42.4 ± 5.5	41.2 ± 7.0	41.5 ± 6.4	40.8 ± 6.1

*Note*: Data are presented as the mean ± SD. A 2 × 2 repeated measures ANOVA was used. Tests of within‐ and between‐subjects effects compared conditions during hypoxia to pre‐ and post‐hypoxia (*p < 0.001) and cervical and thoracic spinal cord injury groups (no significant differences found).

Abbreviations: *f*, breathing frequency; P_ET_CO_2_, end‐tidal CO_2_; SaO_2_, oxygen saturation; *V*_.E._, minute ventilation; *V*_.T._, tidal volume.

For the sham protocol, participants breathed room air for the same amount of time that elapsed during the AIH protocol and at approximately the same time of night. If needed, participants received Zolpidem 30 min before the sleep study on the experimental nights to prevent awakening due to heavy instrumentation to complete the protocol successfully. Three participants with cervical SCI and four with thoracic SCI required the use of Zolpidem. Zolpidem has been used for this purpose in previous studies with SCI participants (Rizwan et al., 2018; Sankari, Bascom, & Badr, 2014; Sankari, Bascom, Chowdhuri, et al., 2014). It has been shown to have minimal or no effect on upper airway collapsibility, ventilation, PaCO_2_, SaO_2_, the ventilatory response to CO_2_, or the respiratory disturbance index (Beaumont et al., 1996; Manning et al., 1992; McCann et al., 1993; Peng & Prabhakar, 2004; Quera‐Salva et al., 1994; Xie et al., 2002). Participants on positive airway pressure (PAP) were held at constant pressure throughout the intervention. The lowest level of PAP that eliminated respiratory events was used. Three participants with cervical SCI and one with thoracic SCI received PAP during the intervention.

### 2.5 Data analysis

The methodology for analyses has also been previously described (Chowdhuri et al., 2015; Sankari, Bascom, et al., 2015). Total breath time, inspiration time, *V*_.T._, P_ET_CO_2_, SaO_2_, and minute ventilation (*V*_.E._) were calculated breath‐by‐breath. For each variable, an average value was determined from a segment of 20 breaths during the control period (a 1‐min segment before the first hypoxia exposure) and during the recovery period (measured at 20, 25, 30, 35, and 40 min after termination of the last hypoxia episode). The presence of ventilatory LTF was determined based on the percent change in *V*_.E._ during the recovery period compared with baseline. Results were compared with those from sham studies. The HVR was calculated based on previously published methods from the AIH periods and was defined as the change in *V*_.E._ for peak ventilation and a corresponding change in SaO_2_ (Δ*V*_.E._/ΔSaO_2_) during each acute hypoxia trial, using nadir hypoxia (between the eupneic breathing just before the hypoxia episode starts and nadir hypoxia; Sankari, Bascom, et al., 2015; Tarbichi et al., 2003). The trace was not shifted back to account for the circulatory delay; however, a large enough segment of breaths was used to capture the desaturation and the recovery for each trial. The first three hypoxia episodes (early HVR) and the last three hypoxia episodes (late HVR) were compared for all subjects and between cervical and thoracic SCI groups to assess the progressive augmentation in response to AIH.

### 2.6 Statistical analysis

The Shapiro–Wilk test was used to assess whether data were normally distributed. Student's independent *t* tests were used to compare participant demographics. A 2 × 2 repeated measures ANOVA in SPSS was used to assess ventilatory parameters (*V*_.E._ *V*_.T._, *f*, SaO_2_, P_ET_CO_2_), using absolute values across study conditions for the AIH (baseline, hypoxia, and recovery) and sham (baseline and recovery) study nights, respectively. The variables used in the model included the study condition (baseline, AIH, and recovery) and cervical and thoracic SCI groups (tests of within‐ and between‐subjects effects). Using procedures for mixed linear modeling in SAS, two different regression models were used to assess the ventilatory response to AIH and are detailed below. A mixed linear model is a generalization of the standard linear model used in the general linear model procedure, the generalization being that the data are permitted to exhibit correlation and nonconstant variability. The mixed linear model, therefore, provides the flexibility of modeling not only the means of the data but their variances and covariances as well. The first mixed linear model was used to test the hypothesis that individuals with cervical SCI would demonstrate potentiated HVR following AIH compared to individuals with thoracic SCI. Specifically, HVR (Δ*V*_.E._/ΔSaO_2_) was assessed using absolute values. The variables used in this model included early and late HVR (within‐subjects effects) and cervical and thoracic SCI groups (between‐subjects effects). The second mixed linear model was used to test the hypothesis that individuals with cervical SCI would demonstrate potentiated LTF following AIH compared to individuals with thoracic SCI. The variables included in this model were recovery from AIH versus corresponding sham exposure (within‐subjects effects) and cervical versus thoracic SCI group (between‐subjects effects) for each ventilatory parameter (*V*_.E._ *V*_.T._, f). Data in this model were entered as a percent of baseline. After stratifying the data by AHI, procedures for mixed linear modeling in SAS were also used to assess whether the severity of SDB influenced the ventilatory response to AIH. A Spearman's correlation test was used to assess the relationship between *V*_.E._ (% of baseline) and AHI and *V*_.E._ (% of baseline) and ODI. A value of *p* < 0.05 was considered to be significant.

## 3 RESULTS

**FIGURE 3:**
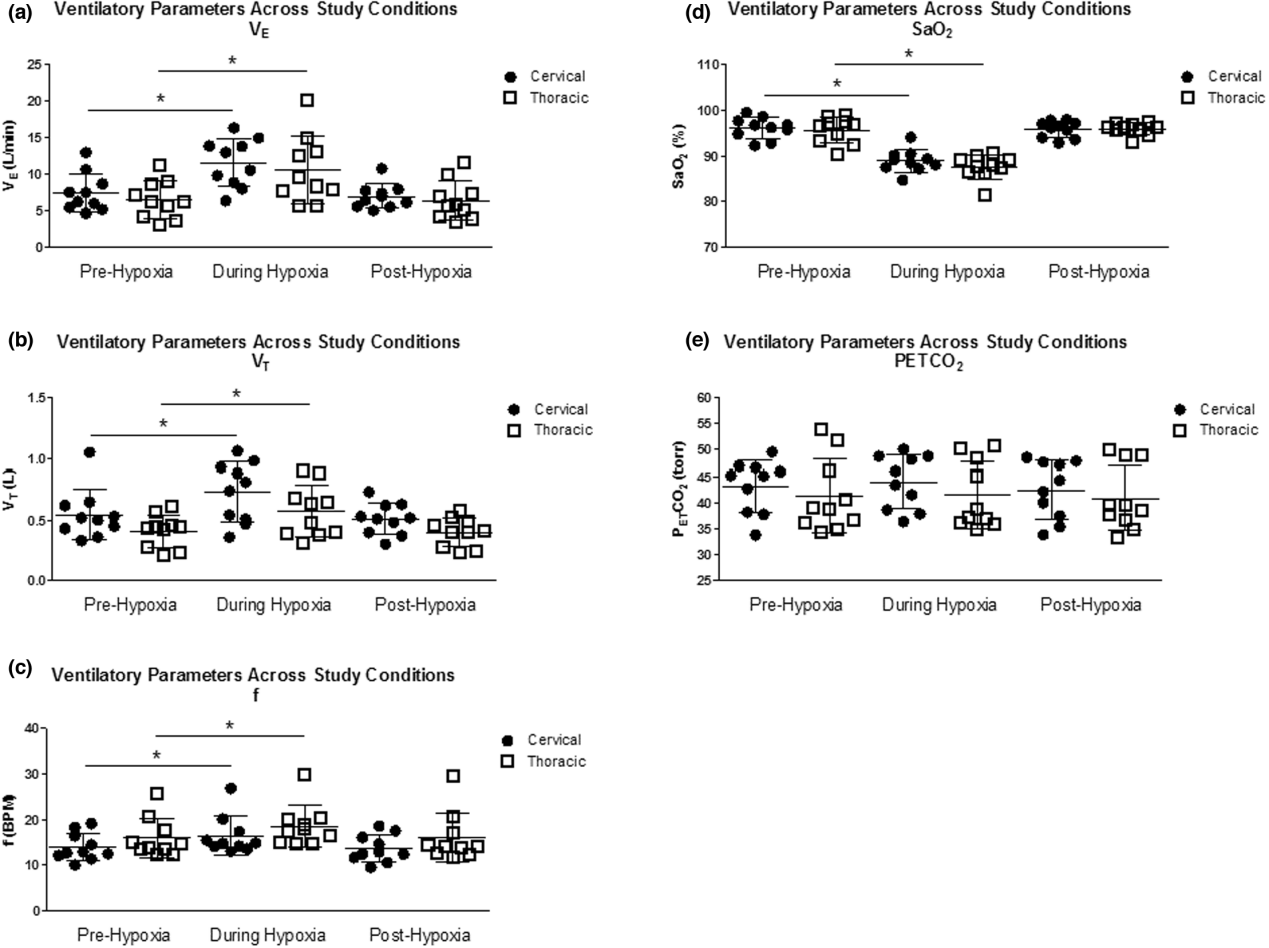
Individual ventilatory parameters across study conditions (pre‐hypoxia, during acute intermittent hypoxia [AIH], and post‐hypoxia), presented as absolute values, in participants with cervical and thoracic SCI. (a) Minute ventilation (*V*_.E._), (b) tidal volume (*V*_.T._), (c) frequency (*f*), (d) oxygen saturation (SaO_2_), and (e) end‐title CO_2_ (P_ET_CO_2_). Data are presented individually and with the group mean ± SD. A 2 × 2 repeated measures ANOVA in SPSS was used to compare differences within and between groups. The variables in this model included study conditions (baseline, AIH, recovery) and cervical and thoracic SCI groups. *Significantly different compared with baseline (*p* < 0.001). SCI, spinal cord injury.

We studied 20 participants with chronic SCI during sleep in the supine position. Ten participants had cervical level injuries (9 male), and 10 had thoracic level injuries (6 male). Participants in the two different groups were similar in age, BMI, AHI, and ODI. Participant characteristics of the group are reported in Table 1. Individual participant characteristics, including age, SCI level, and ASIA score, are presented in Tables 3 and 4. The acute hypoxic effect is displayed in Table 2, showing that during the intervention study, there was a significant change in ventilatory parameters during the AIH compared with baseline (within‐subjects effects; *V*_.E._ [*F* = 38.01, *p* < 0.001], *V*_.T._ [*F* = 17.85, *p* < 0.001], *f* [*F* = 28.03, *p* < 0.001], SaO_2_ [*F* = 97.39, *p* < 0.001]). There was no difference in ventilatory parameters between the two SCI groups (between‐subjects effects; *V*_.E._ [*F* = 0.5, *p* = 0.48], *V*_.T._ [*F* = 3.68, *p* = 0.07], *f* [*F* = 1.40, *p* = 0.25], SaO_2_ [*F* = 0.69, *p* = 0.42], P_ET_CO_2_ [*F* = 0.65, *p* = 0.43]). The levels of P_ET_CO_2_ during hypoxia were not significantly different from pre‐hypoxia and post‐hypoxia in the cervical SCI group (*F* = 1.17, *p* = 0.32) and thoracic SCI group (*F* = 0.65, *p* = 0.43) and demonstrated that isocapnia was maintained during the AIH study condition (Figure 3).

**TABLE 3:** Individual participant demographics with total HVR

Subject	Age	SCI level	ASIA class	Time since injury (years)	Number of hypoxia trials	Total HVR
1	66	SCI‐C6	A	8.3	15	0.27
2	46	SCI‐C6	A	25.4	35	0.88
3	41	SCI‐C6	A	13.6	33	0.62
4	28	SCI‐C6	A	15.6	16	1.29
5	59	SCI‐C6	C	8.2	23	0.61
6	45	SCI‐C6	C	12.1	22	0.71
7	34	SCI‐C6	C	4.8	19	0.88
8	74	SCI‐C5	C	19.4	17	1.41
9	54	SCI‐C4	D	10.7	27	0.41
10	71	SCI‐C	D	24.5	27	0.76
11	41	SCI‐T6	A	11.8	19	0.27
12	37	SCI‐T6	A	3.0	14	ND
13	51	SCI‐T6	A	20.2	30	0.42
14	19	SCI‐T5	A	9.9	15	0.51
15	48	SCI‐T5	A	32.3	23	0.58
16	45	SCI‐T4	A	16.1	28	0.48
17	43	SCI‐T2	A	19.8	15	0.46
18	48	SCI‐T4	B	2.7	25	ND
19	70	SCI‐T6	D	0.8	15	0.23
20	58	SCI‐T3	D	4.7	20	0.63

*Note*: Participant demographics with individual values for total HVR.

Abbreviations: ASIA, American Spinal Injury Association; HVR, hypoxic ventilatory response; ND, not determined; SCI, spinal cord injury.

**TABLE 4:** Individual participant data with ventilatory parameters during the recovery from AIH and corresponding sham

Subject	Age	SCI level	ASIA class	PAP use during study	AIH *V*_.T._ %Control	Sham *V*_.T._ %Control	AIH *V*_.E._ %Control	Sham *V*_.E._ %Control	AIH Freq %Control	Sham Freq %Control
1	66	SCI‐C6	A	Yes	110.69	107.98	108.28	107.24	97.96	98.34
2	46	SCI‐C6	A	No	97.99	94.33	105.45	93.46	92.54	95.42
3	41	SCI‐C6	A	Yes	69.03	93.59	66.07	85.12	95.50	88.48
4	28	SCI‐C6	A	No	98.05	131.61	98.97	146.08	100.78	111.38
5	59	SCI‐C6	C	No	101.98	103.11	105.71	88.40	102.44	85.74
6	45	SCI‐C6	C	No	94.37	60.74	83.11	66.83	91.84	97.05
7	34	SCI‐C6	C	No	90.93	75.12	93.50	102.43	101.80	114.70
8	74	SCI‐C5	C	No	79.10	92.03	82.27	109.96	81.95	117.43
9	54	SCI‐C4	D	No	90.98	82.18	89.29	89.97	98.14	95.08
10	71	SCI‐C	D	Yes	105.10	89.10	98.39	108.37	94.08	105.30
11	41	SCI‐T6	A	No	90.83	62.94	83.29	78.42	91.00	111.33
12	37	SCI‐T6	A	No	84.76	87.31	81.34	67.22	95.14	75.82
13	51	SCI‐T6	A	No	89.02	64.37	98.90	76.03	112.71	104.43
14	19	SCI‐T5	A	No	101.91	105.46	108.30	108.26	106.50	102.62
15	48	SCI‐T5	A	No	76.30	34.99	86.36	46.62	115.16	124.58
16	45	SCI‐T4	A	No	82.91	111.19	78.47	119.43	93.83	106.19
17	43	SCI‐T2	A	No	97.99	78.79	106.88	100.13	96.69	110.92
18	48	SCI‐T4	B	Yes	75.30	84.27	65.01	90.86	86.76	107.06
19	70	SCI‐T6	D	No	98.23	84.10	97.74	80.12	101.27	96.19
20	58	SCI‐T3	D	No	113.92	105.32	117.00	114.17	102.29	107.33

*Note*: Participant demographics with individual ventilatory changes presented as the average (%) change from baseline in *V*_.T._, *V*_.E._, and Freq compared between the 20‐ and 40‐min period of the recovery following AIH or corresponding sham.

Abbreviations: AIH, acute intermittent hypoxia; ASIA, American Spinal Injury Association; Freq, breathing frequency; PAP, positive airway pressure; SCI, spinal cord injury; *V*_.E._, minute ventilation; *V*_.T._, tidal volume.

**FIGURE 4:**
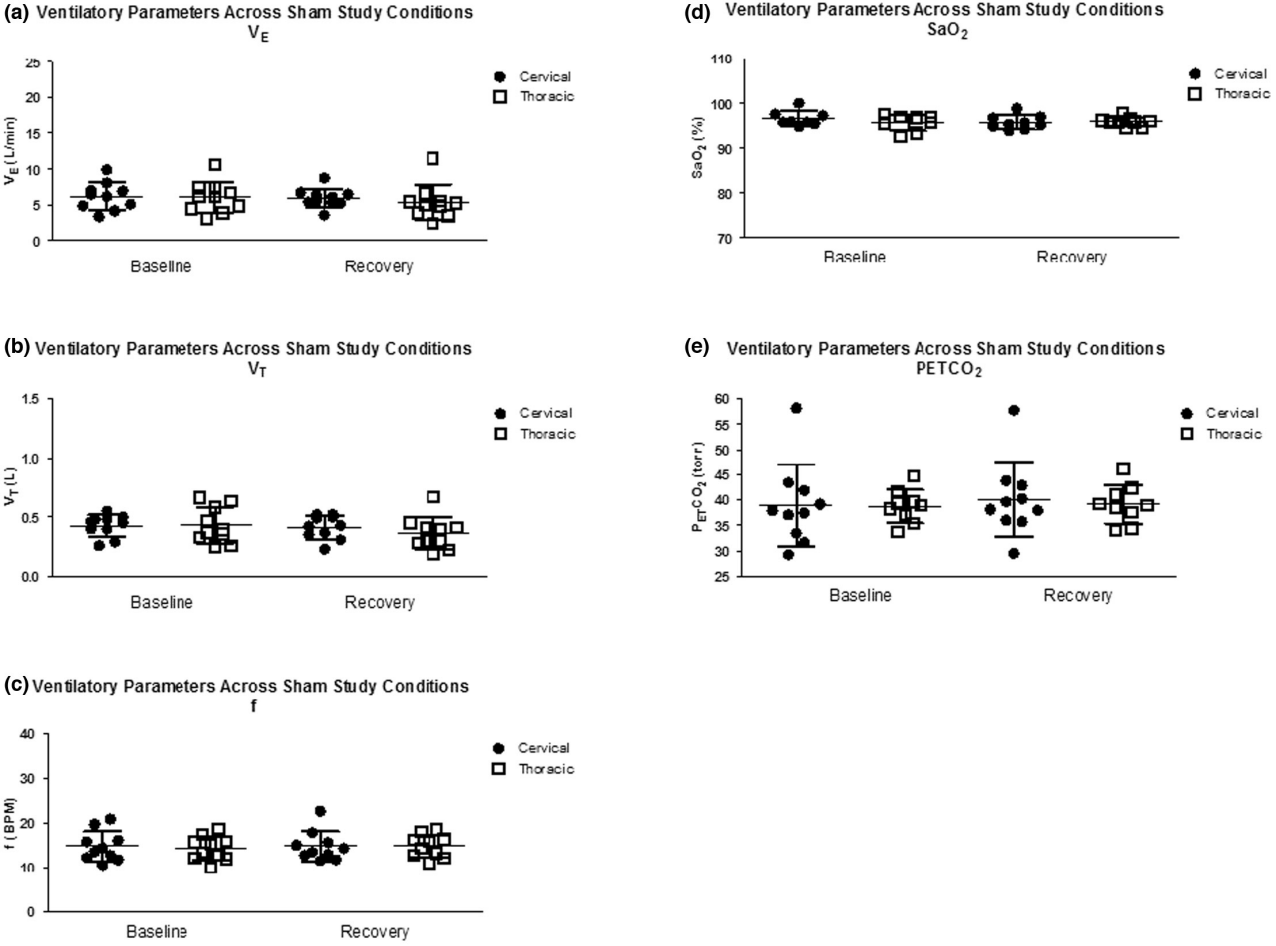
Individual ventilatory parameters across sham study conditions (baseline and recovery), presented as absolute values, in participants with cervical and thoracic SCI. (a) Minute ventilation (*V*_.E._), (b) tidal volume (*V*_.T._), (c) frequency (*f*), (d) oxygen saturation (SaO_2_), and (e) end‐title CO_2_ (P_ET_CO_2_). Data are presented individually and with the group mean ± SD. A 2 × 2 repeated measures ANOVA in SPSS was used to compare differences within and between groups. The factors used in the model included study condition (baseline and recovery) and cervical and thoracic SCI groups. No significance or interactions were found between or within groups. SCI, spinal cord injury.

Under sham conditions, there was no difference in ventilatory parameters between the cervical and thoracic SCI groups (*V*_.E._ [*F* = 0.18, *p* = 0.68], *V*_.T._ [*F* = 0.24, *p* = 0.63], *f* [*F* = 0.03, *p* = 0.87], SaO_2_ [*F* = 0.40, *p* = 0.54], P_ET_CO_2_ [*F* = 0.05, *p* = 0.83]) or between the baseline and recovery periods (*V*_.E._ [*F* = 2.2, *p* = 0.16], *V*_.T._ [*F* = 3.45, *p* = 0.08], *f* [*F* = 0.45, *p* = 0.51], SaO_2_ [*F* = 1.7, *p* = 0.21], P_ET_CO_2_ [*F* = 1.11, *p* = 0.31]). Ventilatory parameters for the sham protocol are presented in Figure 4.

### 3.1 Ventilatory responses to hypoxia (short‐term ventilatory response)

**FIGURE 5:**
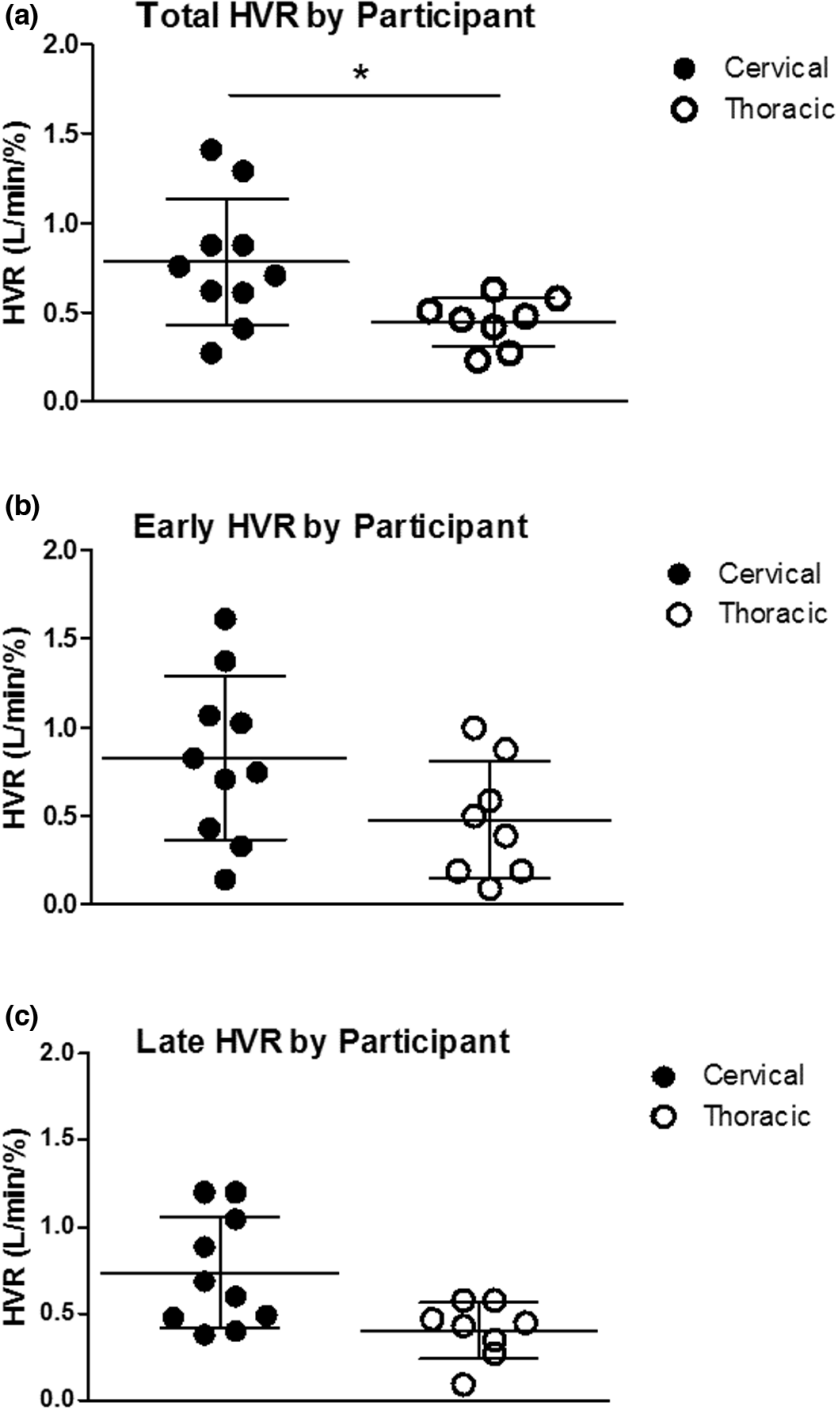
Individual data to illustrate hypoxic ventilatory response (HVR) during acute intermittent hypoxia episodes in participants with cervical (*n* = 10) and thoracic (*n* = 8) SCI. (a) Total HVR, (b) early HVR episodes, and (c) late HVR episodes. Data are presented as the mean ± SD. Mixed linear modeling in SAS was used. The variables used in this model included early and late HVR and cervical and thoracic SCI groups. *Significantly different compared with cervical *p* < 0.05. SCI, spinal cord injury.

Exposure to hypoxia resulted in a significant increase in minute ventilation (*V*_.E._), tidal volume (*V*_.T._), and respiratory frequency (*f*) compared to baseline in both cervical and thoracic SCI groups (*p* < 0.001; Table 2; Figure 3). HVR was determined for 18 of the 20 participants. HVR could not be determined for two of the thoracic SCI participants as the hypoxia episodes destabilized their breathing to a point at which consistent signals could not be obtained in the nonhypoxic period of the episodes. There was a significant difference in total HVR (Δ*V*_.E._/ΔSaO_2_) between the two groups (cervical [0.78 ± 0.35 L/min/%] vs. thoracic [0.45 ± 0.14 L/min/%]; SCI, *p* = 0.023), as depicted in Figure 5. However, there was no difference between early HVR (cervical SCI [0.83 ± 0.46 L/min/%]; thoracic SCI [0.48 ± 0.33 L/min/%]) and late HVR (cervical SCI [0.74 ± 0.32 L/min/%]; thoracic SCI [0.40 ± 0.16 L/min/%] *p* = 0.37) and no interaction with time or SDB severity (*p* = 0.94). Figure 5 depicts the acute hypoxic effect. As there is no sham equivalent, sham data are not presented. Individual data for total HVR are presented in Table 3.

### 3.2 Effect of AIH on ventilation after the hypoxic exposure (long‐term ventilatory response)

**FIGURE 6:**
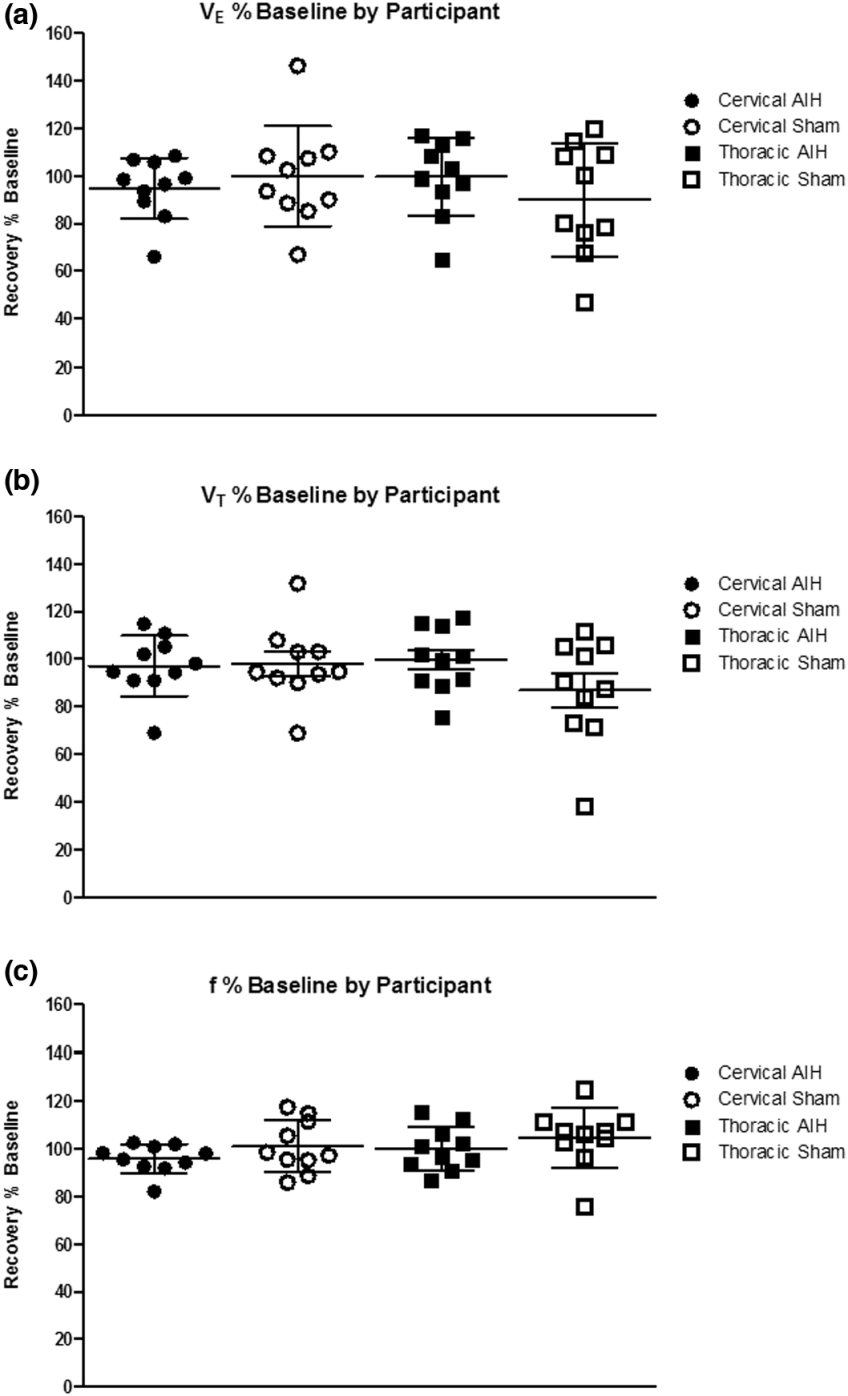
Ventilatory changes presented as the average (%) change from baseline in (a) minute ventilation (*V*_.E._), (b) tidal volume (*V*_.T._), and (c) frequency (*f*) for individual participants grouped by injury level (cervical or thoracic SCI) and recovery from AIH or corresponding sham protocol. Individual data are presented for each participant with the group mean ± SD. Mixed linear modeling in SAS was used. The variables used in the model included recovery from AIH versus corresponding sham exposure and cervical and thoracic SCI groups (within‐subjects, between groups, and within‐subjects by between‐group interaction). No statistically significant differences were found. AIH, acute intermittent hypoxia; SCI, spinal cord injury.

Following exposure to AIH, there was no significant difference in *V*_.E._, *V*_.T._, or respiratory frequency from baseline during the recovery period (between 20 and 40 min) when comparing either the AIH and sham studies (*p* = 0.69, 0.20, 0.13, respectively) or the cervical SCI and thoracic SCI groups (*p* = 0.70, 0.46, 0.22, respectively; Figure 6). Individual data for each of the ventilatory parameters are presented in Table 4. To determine the potential determinants of *V*_.E._ in the recovery period, we used Spearman's correlation analysis to determine a relationshi*p* between *V*_.E._ and AHI and/or *V*_.E._ and ODI. There was no correlation between *V*_.E._ in the recovery period and AHI (*R* = −0.22, *p* = 0.34) or ODI (*R* = 0.13, *p* = 0.58). To determine whether the severity of SDB influenced the ventilatory response to AIH, we analyzed the data stratifying by the severity of SDB (≥15 and <15) and still found no evidence of an increase in ventilatory parameters (*V*_.E._ [*p* = 0.14], *V*_.T._ [*p* = 0.11], and *f* [*p* = 0.63]) during the recovery phase after AIH.

## 4 DISCUSSION

Our study demonstrated the following findings regarding the effects of AIH on respiratory plasticity during sleep in individuals with chronic SCI: (1) Ventilatory response to isocapnic hypoxia during sleep significantly increased in cervical SCI compared to thoracic SCI participants and (2) Compared to sham, there was no significant increase in *V*_.E._ and *V*_.T._ (presented as the average and % change from baseline) during the recovery phase after AIH in cervical or thoracic SCI participants.

### 4.1 HVR during sleep in chronic SCI

The HVR is a recognized manifestation of respiratory plasticity following exposure to acute episodic hypoxia (Mateika & Komnenov, 2017). We noted that exposure to AIH was associated with increased HVR only in participants with cervical SCI. The increased HVR in cervical SCI indicates enhanced peripheral chemo‐responsiveness following episodic hypoxia (Tarbichi et al., 2003). The difference in HVR between people with cervical and thoracic injuries in sleep is likely due to decreased HVR (nearly 50%) in thoracic SCI but not cervical in non‐REM sleep. This decrease in HVR between wakefulness and sleep in thoracic SCI corroborate previous studies in able‐bodied individuals (Douglas et al., 1982). This contrasts with a recent study showing no difference in HVR during wakefulness when comparing cervical versus thoracic SCI (Sankari, Bascom, et al., 2015). The increased HVR in participants with cervical SCI during sleep indicate increased peripheral chemosensitivity to hypoxia which may contribute to the pathogenesis of SDB in this population.

### 4.2 LTF during sleep in chronic SCI

Ventilatory LTF is a form of respiratory plasticity following AIH and is characterized by a sustained increase in minute ventilation over time. The intervention protocol in the current study was designed to control the dose and duration of AIH and mimic moderate SDB severity. The lack of hyperpnea during the recovery period in individuals with SCI following AIH indicates the absence of ventilatory LTF, a finding contrary to previous studies in individuals with SCI during wakefulness (Olson et al., 2001; Sankari, Bascom, et al., 2015). A similar lack of LTF was noted in patients with obstructive sleep apnea and older adults (Aboubakr et al., 2001; Chowdhuri et al., 2008) during NREM sleep. In contrast, ventilatory LTF following AIH has been previously observed in young, healthy adults and animals during sleep (Babcock et al., 2003; Chowdhuri et al., 2008, 2010; Pierchala et al., 2008; Shkoukani et al., 2002). A study of LTF following AIH by Vermeulen et al. (2020) found that males and females developed similar LTF through different respiratory recruitment patterns. Their participants included healthy young individuals with a mean age of 22 ± 3 years. A previous study by our group demonstrated no difference in the ventilatory response to hypocapnic AIH between males and females (Tarbichi et al., 2003); therefore, gender differences in this study should not have affected the results. The unequal distribution of males and females in the current study is listed as a limitation below. A study by Chowdhuri et al. (2015) comparing the effect of AIH during NREM sleep on younger versus older adults (mean age of older vs. younger adults was 62 ± 8 and 26.4 ± 4.3, respectively) found an absence of ventilatory LTF following AIH in older adults. LTF was present in the young adults and an increase in HVR compared with the older adults. The study by Chowdhuri et al. (2015) also found similar results between males and females. These results suggest a role for age in the development of LTF following AIH but not gender. With regard to age, our participants fall between the two groups and have a larger span than those in the studies mentioned above, with a mean age of 48.9 ± 14.5. These differing results suggest variable manifestations of ventilatory LTF across different groups and different experimental paradigms.

Another important observation was the lack of significant differences in *V*_.E._ in the recovery period following AIH in cervical and thoracic SCI. This blunting of ventilatory changes during recovery in both SCI levels suggests that the level of injury was not a significant determinant of ventilatory LTF during sleep in individuals with chronic SCI. Results show a decrease in *V*_.E._ during recovery periods compared with baseline for several of the participants. This experiment cannot fully explain the observed changes in *V*_.E._ between recovery and baseline. However, the noted decrease in *V*_.E._ during recovery periods was mainly driven by reduced *V*_.T._ in both AIH and sham nights. A plausible physiological explanation would be the inhibited ventilatory motor outputs under low serotonin (a pivotal factor to generate respiratory plasticity) in highly susceptible individuals with SDB (Ling et al., 2001). Furthermore, studies showed that both serotonin receptors and adenosine receptors are activated during AIH. Adenosine receptors may constrain the expression of serotonin‐dependent phrenic and hypoglossal LTF following AIH. In fact, adenosine A2A receptor antagonists (such as wake‐promoting substance caffeine) were able to enhance AIH‐induced respiratory plasticity. Hence, the predominance of adenosine activity during sleep may exert a deleterious effect on LTF (Hoffman et al., 2010).

The paradoxical absence of ventilatory LTF during sleep, despite a robust ventilatory LTF during wakefulness, was intriguing and counter to previous studies where ventilatory LTF is more likely to occur during sleep than during wakefulness. Nevertheless, ventilatory LTF following episodic hypoxia is not universal and is affected by many variables, including experimental paradigm, species, or even rats' strain (Baker‐Herman et al., 2010). Methodological differences are unlikely to explain our findings since we used the same experimental protocol in our previous LTF studies and maintained isocapnia during the hypoxic exposure periods. One possible explanation is whether the presence of SDB dampens ventilatory LTF. A previous study from our laboratory demonstrated the absence of ventilatory LTF during NREM sleep following AIH in patients with obstructive sleep apnea (Aboubakr et al., 2001). Likewise, ventilatory LTF is significantly diminished in rats following 7 days of CIH (Edge & O'Halloran, 2015). The lack of ventilatory LTF after exposure to CIH mimics SDB and may explain our findings in participants with chronic SCI who have moderate to severe SDB. Another potential explanation is SDB‐induced serotonin depletion during sleep. Thus, blunting of ventilatory LTF in the presence of SDB may represent a compensatory mechanism for enhanced phrenic motoneurons after chronic cervical SCI (Fuller et al., 2003), potentially stabilizing the neural function following repetitive perturbations (Braegelmann et al., 2017; Turrigiano, 2012).

### 4.3 Methodological considerations

Several considerations may influence the interpretation of our findings. First, the presence of hypocapnia can dampen the magnitude of LTF. Therefore, supplemental CO_2_ was applied to maintain isocapnia acutely during hypoxia episodes and prevent the possible dampening effect of hypocapnia. This is the same paradigm of supplemental CO_2_ that is used in our previous LTF studies. Therefore, we do not believe that hypocapnia was responsible for the absence of LTF since our hypoxic episodes were either isocapnic or slightly hypercapnic. Second, ventilatory changes related to hypoxia in cervical SCI could not be due to time‐related ventilatory changes, given the same procedure, and time control was also used in thoracic SCI. Thus, ventilatory augmentation was likely due to increased peripheral ventilatory responsiveness in individuals with cervical SCI. Third, Zolpidem was used in some participants from both groups but was not used consistently between the experimental and sham visits as the sham visits were combined with the PSG. However, as mentioned in the Methods section under “Measurements,” Zolpidem has been shown to have minimal or no effect on upper airway collapsibility and ventilation; however, some studies have shown that Zolpidem may lower oxygen saturation (Gatti et al., 2016). Fourth, two participants in each group (cervical and thoracic) used full‐face masks, whereas all other participants used nasal masks. We think it unlikely that differences in mask type influenced the results as both types of masks are effective when mouth breathing is prevented and CO_2_ is monitored (Willson et al., 2004). Finally, the study did not include an equal number of males and females in each group. While this would be ideal and enhance the findings from this study, SCI participants are difficult to recruit, and SCI disproportionately affects males compared with females. However, as mentioned above, previous studies have not demonstrated a difference in LTF following AIH in males compared with females (Chowdhuri et al., 2015; Vermeulen et al., 2020).

### 4.4 Clinical implications

Our findings have several potential clinical implications related to the pathogenesis and treatment of SDB in individuals with cervical compared with thoracic SCI. First, the absence of significant ventilatory LTF during sleep in people living with SCI eliminates one potentially protective mechanism that maintains upper airway patency and enhances stable breathing during sleep. Second, the increase in HVR may promote central apnea and further destabilize respiration. This observation may provide a physiologic explanation for the high frequency of recurrent central apneas and underscore the negative impact of recurrent hypoxia in this population. There is a need to ascertain whether amelioration of CIH with supplemental Oxygen could promote long‐term breathing stability. About 50% of patients with central apnea respond to positive airway pressure therapy (CPAP), while approximately 50% require additional therapies such as oxygen or adaptive servo‐ventilation (Chowdhuri et al., 2012). Third, the selective response to AIH during wakefulness may inform studies investigating the role and timing of therapeutic AIH. Specifically, our findings suggest that delivering therapeutic AIH may be more efficacious during wakefulness than during sleep (Vivodtzev et al., 2020).

In summary, we have found that hypoxic chemo‐responsiveness is significantly increased during AIH in the cervical group compared with the thoracic group. However, these changes in ventilation ceased immediately after the termination of hypoxia, with no evidence of ventilatory LTF during the recovery from hypoxia in either the cervical or thoracic groups.

## AUTHOR CONTRIBUTIONS

Conception and design: Abdulghani Sankari and M. Safwan Badr. Analysis and interpretation: Sarah Vaughan, Abdulghani Sankari, Sean Carroll, Mehdi Eshraghi, Harold Obiakor, Hossein Yarandi, Susmita Chowdhuri, Anan Salloum, and M. Safwan Badr. Drafting the manuscript: Sarah Vaughan, Abdulghani Sankari, and M. Safwan Badr. Creating figures: Sarah Vaughan, Sean Carroll, and Mehdi Eshraghi.

## FUNDING INFORMATION

Dr. Badr was supported by the Department of Defense #SC150201, Department of Veterans Affairs, Merit Review #1I01CX001040, and National Heart, Lung, Blood Institute #R01HL130552. Dr. Sankari was supported by a Career Development Award from the (U.S.) Department of Veterans Affairs Office of Research and Development #IK2CX000547, #RX002885, Department of Defense #SC150201, and from the National Heart, Lung, and Blood Institute Awards #HL140447 and #HL130552. Dr. Chowdhuri was supported by the Department of Veterans Affairs, Clinical Science Research, and Development Award #1I01CX001201‐01A1.

## CONFLICT OF INTEREST

The authors declared that they have no conflict of interest.

## DISCLOSURE

This was not an industry‐supported study. This article's content is solely the authors' responsibility and does not represent the views of the Department of Veterans Affairs, NIH, or the United States government.

## ETHICS STATEMENT

The Human Investigation Committees of the Wayne State University School of Medicine and the John D. Dingell Veterans Affairs Medical Center approved the experimental protocols.

## PATIENT CONSENT

Participants provided written informed consent prior to completing any study activities.

## CLINICAL TRIAL REGISTRATION

This study was registered on Clinicaltrials.gov under NCT02922894.
